# Laparoscopic resection of a large clinically silent paraganglioma at the organ of Zuckerkandl: a rare case report and review of the literature

**DOI:** 10.1186/s12894-020-00732-0

**Published:** 2020-10-07

**Authors:** Xiang Ren, Jiwen Shang, Ruimin Ren, Huajun Zhang, Xue Yao

**Affiliations:** 1 Graduate School, Shanxi Medical University, Taiyuan, 030000 China; 2 Department of Urology, Shanxi Bethune Hospital, No. 99 Longcheng Street, Taiyuan, 030032 Shanxi China

**Keywords:** Paraganglioma, Large, Silent, Organ of zuckerkandl, Laparoscopy, Genetic testing

## Abstract

**Background:**

Large paraganglioma of the Zuckerkandl organ (POZ) is extremely rare. The patient can occasionally be paucisymptomatic, further obscuring the diagnosis and carrying high mortality. Recommended treatment for large paraganglioma (PGL) is open surgical removal. We report a case of successful laparoscopic resection of a large POZ with normal blood pressure in a 45-year-old man.

**Case presentation:**

A 45-year-old man was hospitalized because of hyperglycemia. Computed tomography of the abdomen and the serum and urinary catecholamine levels confirmed the diagnosis of large POZ. But his blood pressure was normal and he underwent laparoscopic tumor excision successfully. During 6 months follow-up after laparoscopy, serum and urinary catecholamines were normal but glycaemia remained high level. DNA analysis of the succinate dehydrogenase complex subunits B (SDHB) and SDHD revealed no mutation.

**Conclusions:**

POZ is an unusual mass and preoperative diagnosis can be difficult in clinically silent cases. PGL cannot be excluded in patients with normal blood pressure. Even a large POZ can be excised laparoscopically by following proper techniques.

## Background

PGLs are catecholamine-producing tumors that may occur anywhere along the sympathetic paraganglionic chain [1]. The treatment of PGL including its diagnosis is difficult, especially when some patients lack typical clinical signs and symptoms (headache, palpitations, sweating, or hypertension) which are due to the direct actions of secreted catecholamines. About 53% of PGLs occur in the organs of Zuckerkandl [2], which are a collection of paraganglia located anterolaterally to the distal abdominal aorta between the origin of the inferior mesenteric artery or renal artery and the aortic bifurcation. The majority of POZs are non-functional [3], so misdiagnosis and missed diagnosis are common. Traditional treatment for large POZ was open surgical resection, and only a few cases of laparoscopic approach to this pathology have been reported (Table 1). We report a case of successful laparoscopic resection of a large POZ with normal blood pressure in a 45-year-old man and review the related literature on POZ.

**Table 1:** Cases of laparoscopic resection of a large POZ reported in the literature

Case	References	Misdiagnosis	Age (years)	Size (cm)	Time (min)	Blood loss (mL)	Genetic screening	Gene mutation
1	Kravarusic et al. [4]	No	14	6	NA	NA	Yes	SDHB
2	Thapar et al. [5]	No	20	6.5 × 5	NA	300	No	–
3	Cozzupoli et al. [6]	NA	20	10	NA	NA	NA	NA
4	Salgaonkar et al. [3]	No	22	8 × 7	125	40	No	–
5	Joshi et al. [7]	Yes	28	7.5 × 6.6 × 7.5	NA	NA	No	–
Present		Yes	45	7.2 × 6.5	120	50	Yes	No

*NA* not available

## Case presentation

**Fig. 1:**
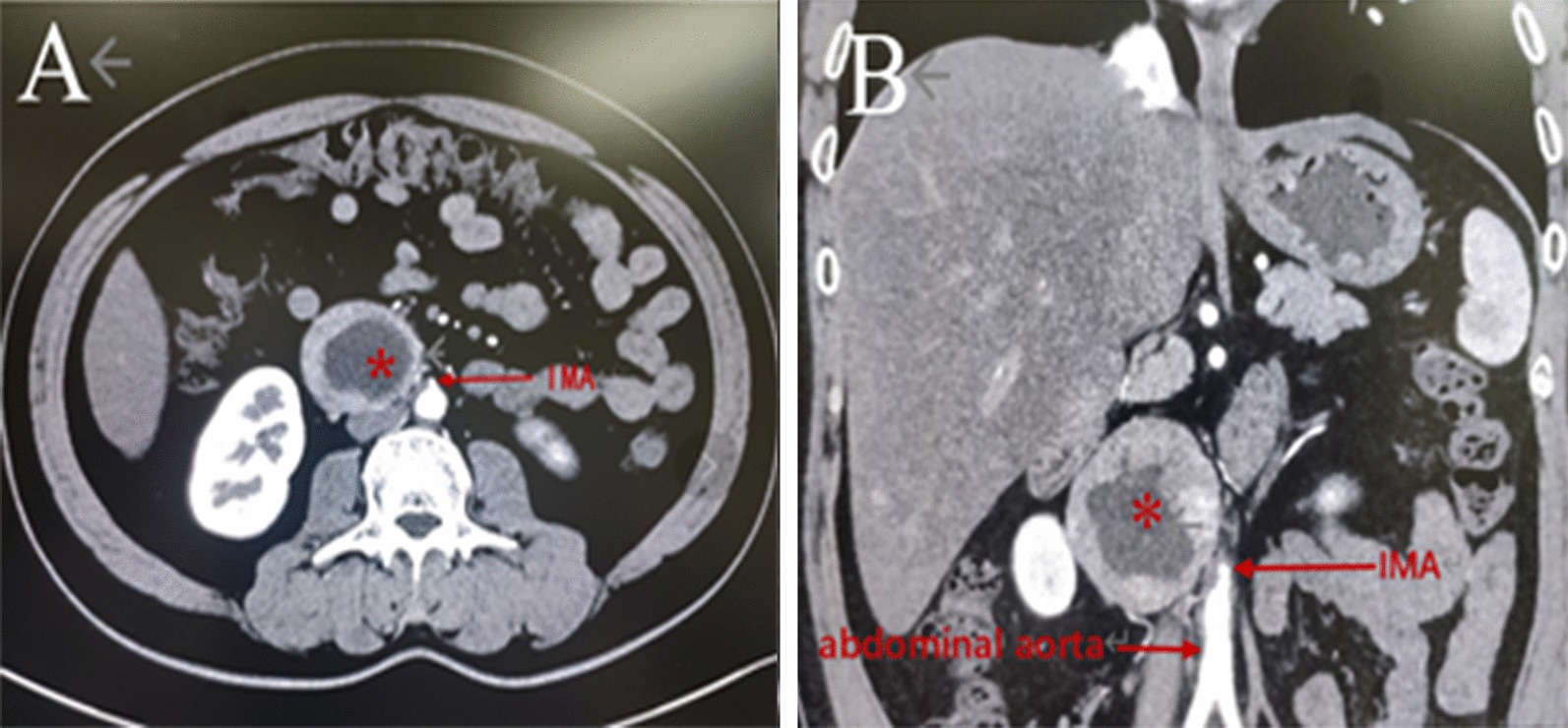
CT findings: CT shows a 7.2 × 6.5 cm right para-aortic mass (asterisk) located at the level of the inferior mesenteric artery (IMA). Transverse plane (**a**). Coronal plane (**b**)

A 45-year-old man with a half-year history of hyperglycemia presented to endocrinology. The patient denied attacks of headache, hypertension, palpitation and sweating. He was kept for observation with 24-h electrocardiography (ECG) and blood pressure monitoring which was normal. His glycaemia returned to normal level under the action of insulin. Haematological and biochemical investigations were normal except for elevated serum and urinary catecholamines (Table 2). Abdominal-pelvic CT scan with contrast injection had revealed a 7.2 × 6.5 cm inhomogeneous, right para-aortic mass located at the level of the inferior mesenteric artery (Fig. 1). Both the adrenals were normal. With these findings, a diagnosis of POZ was reached. However, due to the patient's family economic reasons and the lack of MIBG scan examination equipment in our hospital, our patient did not undergo further MIBG scan examination. Then he was transferred to our center where attempts to preoperative preparation included expansion of intravascular volume and alpha blockade with phenoxybenzamine for 2 weeks even with the situation of normal blood pressure.

**Table 2:** Blood and urinary analysis

	Upon presentation	6 months follow-up	Reference values
Blood pressure (max)	118/76 mmHg	115/75 mmHg	100–120/60–80 mmHg
Fasting blood-glucose (max)	7.5 mmol/L	7.4 mmol/L	3.9–6.1 mmol/L
*Blood*			
Adrenaline	1050 pg/mL	39.7 pg/mL	0–100 pg/mL
Noradrenaline	650 pg/mL	168.0 pg/mL	0–600 pg/mL
Dopamine	102 pg/mL	59.17 pg/mL	0–100 pg/mL
*Urine*			
Metanephrines 24 h	2120 mg/24 h	190 mg/24 h	64–350 mg/24 h
Normetanephrines 24 h	4252 mg/24 h	260 mg/24 h	120–490 mg/24 h
Vanillylmandelic acid 24 h	35 mg/24 h	6.0 mg/24 h	2.0–7.1 mg/24 h

We made full consideration and preparation for possible conversion to open surgery before deciding to adopt laparoscopic surgery. If there were acute bleeding, small operation space, malignant tumor invasion or other parts of the injury that have to be converted to open surgery, we will use a longitudinal incision under the right costal margin for open surgery. In addition, we also communicated with vascular surgeons that if the aorta or the inferior vena cava were damaged, open resection and reconstruction of these vessels might be warranted.

**Fig. 2:**
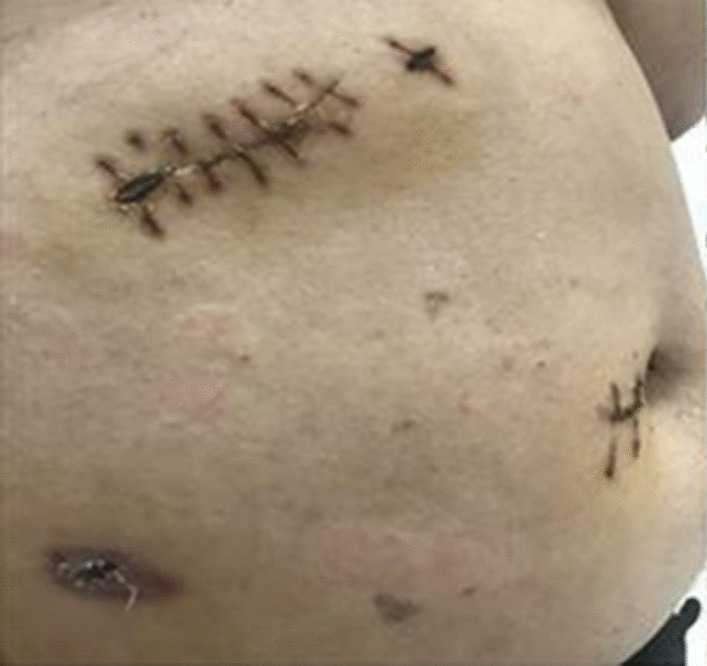
Positions of the ports. A 10-mm trocar was inserted above the umbilicus, and another 10-mm, 10-mm and 5-mm trocar were placed under the right costal margin, right anterior axillary line and subxiphoid, respectively

The patient was placed in 70° left lateral decubitus position, and a 10-mm trocar was inserted above the umbilicus for telescope using. Another 10-mm, 10-mm and 5-mm trocar were placed under the right costal margin, right anterior axillary line and subxiphoid, respectively (Fig. 2). A steep Trendelenburg position allowed the small bowel and omentum to be moved away from the mass. First, cut the hepatic triangular ligament and hepatocolicum ligament with an ultrasonic shears, and lift the liver to expose the upper pole of the right kidney. The ascending mesocolon is then longitudinally incised and pushed open to expose the right kidney and the right renal vein, and the descending and horizontal parts of the duodenum are separated and pushed open, showing that the upper pole of the exposed tumor is filled with nourishing vessels. Then the vessels of nutritional tumors were separated and ligated one by one. Uncovering the lower part of the tumor, it can be seen that the inferior vena cava and abdominal aorta, and the abdominal aorta send out a thicker vascular supply tumor, which was ligated by Hem-o-lock. The tumor and its surrounding tissues were then clipped along the tumor capsule and separated gradually. The well-encapsulated mass was excised. The mass was placed in a sample collection bag and removed through a 5-cm right upper abdomen incision that incorporated the incision made for the costal margin 10-mm port. Except for upon contact with the tumor blood pressure rose up to 160 mmHg he remained stable throughout surgery. The total operative time was 120 min and intraoperative blood loss was around 50 mL. The post-operative period was uneventful and he was discharged on post-operative day 5. The histopathology found the tissue was consistent with a benign PGL and DNA analysis of SDHB and SDHD revealed no mutation. During 6 months follow-up after post-operative, urinary and serum catecholamines were within normal range but glycaemia remained high level (Table 2).

## Discussion and conclusion

PGLs are rare catecholamine-secreting neuroendocrine tumors that arise from the extra-adrenal paraganglia. Most PGLs occur in the subdiaphragmatic region, most commonly within the organ of Zuckerkandl [8]. The clinical presentation of pheochromocytomas/PGLs can vary remarkably, from dramatic symptoms and signs to minimal or no symptoms whatsoever [9]. This latter situation is often termed “clinically silent” pheochromocytoma/PGL [10, 11]. Even though the patient with a clinically silent PGL experiences no symptoms and shows no signs of a PGL, blood and urine levels of catecholamines are often elevated [10–12]. This situation usually leads to misdiagnosis and missed diagnosis. Therefore, understanding its clinical characteristics is very important for the standardized diagnosis and treatment of clinical diseases. In our case, the patient without characteristic paroxysmal attacks of headache, sweating, palpitation and hypertension only presented with hyperglycemia. Although pheochromocytomas/PGLs can cause hyperglycemia and even induce diabetes type 2 [13], the patient's glycaemia remained high level at 6-month follow-up after post-operative when urinary and serum catecholamines were normal. It confirmed that this hyperglycemia was presumably not related to the POZ, but merely coincided by chance.

A giant PGL/pheochromocytoma usually present with paucity of clinical signs and symptoms [14, 15]. The mechanism of clinically silent PGL/pheochromocytoma remains unclear. The reasons for the same can be due of the presence of tumoral necrosis, high loads of interstitial tissue compared to chromaffin cells or the paucity of the release of the catecholamines due to encapsulation by the connective tissues [15]. These cause normal catecholamine values during their serum and urinary assays [16]. There are also studies that believe that there is a self-metabolism mechanism of catecholamines inside the tumor, which prevents active catecholamines from being released into the blood [17]. These explanations are not consistent with our case. Therefore, the mechanism of this situation needs to be further explored in subsequent studies.

Previously, it was considered that there were no guidelines to ensure adequate preoperative preparation in these clinically silent tumors [10]. The present view holds that patients suspected of clinically silent pheochromocytoma should be given a high sodium diet and adequate fluid intake before surgery, and alpha blockade should be prepared for 7–14 days to avoid possible cardiovascular damage caused by abrupt blood pressure rise and fall and insufficient blood volume during and after surgery [18]. Surgical resection is the treatment of choice for POZ, and laparoscopic resection is suited for small (< 7 cm) tumors [3]. Only eight cases of laparoscopic resection of POZ have been described till to 2020. Depending on their location, strategies utilized to approach the tumor included duodenal Kocherisation as well as colonic mobilization [3]. Under the situation of our patients with tumors > 7 cm, we chose laparoscopic resection with duodenal Kocherisation, exposed the tumor directly after freeing the kidney, and early controlled the blood vessels from the abdominal aorta to reduce intraoperative bleeding and minimize intraoperative hemodynamic instability.

Most PGLs appear to be sporadic; however, about 30–35% of tumors are associated with hereditary syndromes, mainly multiple endocrine neoplasia type 2 (MEN-2), von Hippel Lindau (VHL) disease, and neurofibromatosis type 1(NF1) [19]. 70% of POZ cases are associated with SDHB or less commonly SDHD gene mutations [20]. In our case, blood calcitonin, serum free T3, free T4, and TSH levels were normal. These results make MEN-2 disease unlikely. Ophthalmological screening showed an absence of retinal hemangioblastoma that makes VHL disease unlikely. The lack of cutaneouscafé-au-lait spots suggested the absence of NF1. DNA analysis of SDHB and SDHD revealed no mutation. In addition, genetic testing should be performed for at-risk family members [21].We once advised his family members to do the related genetic tests, but they refused due to the cost. Nonetheless, a predisposing genetic factor cannot be excluded since current research has revealed several other genes linked to familial and sporadic cases of pheochromocytoma [22]. Thus, we advised the patient to return for regular follow-up.

In conclusion, POZ is an unusual mass and preoperative diagnosis can be difficult in clinically silent cases. PGL cannot be excluded in patients with normal blood pressure. Even a large POZ can be excised laparoscopically by following proper techniques.

## Data Availability

All data supporting our findings are contained within the manuscript.

## Abbreviations

POZ: Paraganglioma of the Zuckerkandl organ
PGL: Paraganglioma
CT: Computed tomography
SDHB: Succinate dehydrogenase complex subunit B
SDHD: Succinate dehydrogenase complex subunit D
ECG: Electrocardiography
Max: Maximum
IMA: Inferior mesenteric artery
MEN-2: Multiple endocrine neoplasia type 2
VHL: Von Hippel Lindau
NF1: Neurofibromatosis type 1
TSH: Thyroid stimulating hormone
